# Neural stem cells derived from α-synuclein-knockdown iPS cells alleviate Parkinson’s disease

**DOI:** 10.1038/s41420-024-02176-z

**Published:** 2024-09-17

**Authors:** Chie-Hong Wang, Guan-Cyun Lin, Ru-Huei Fu, Yu-Chuen Huang, Shih-Yin Chen, Shinn-Zong Lin, Horng-Jyh Harn, Woei-Cherng Shyu, Yi‐Fang Huang, Long-Bin Jeng, Shih-Ping Liu

**Affiliations:** 1 Cell Therapy Center, China Medical University Hospital, Taichung, 404 Taiwan; 2 Neuroscience and Brain Disease Center, College of Medicine, China Medical University, Taichung, 411 Taiwan; 3 Department of Neurology, China Medical University Hospital, Taichung, 404 Taiwan; 4 Graduate Institute of Biomedical Science, China Medical University, Taichung, 411 Taiwan; 5 Ph.D. Program for Aging, College of Medicine, China Medical University, Taichung, 411 Taiwan; 6 Translational Medicine Research Center, China Medical University Hospital, Taichung, 404 Taiwan; 7 Genetics Center, Department of Medical Research, China Medical University Hospital, Taichung, 404 Taiwan; 8 School of Chinese Medicine, College of Chinese Medicine, China Medical University, Taichung, 411 Taiwan; 9 Buddhist Tzu Chi Bioinnovation Center, Buddhist Tzu Chi Medical Foundation, Hualien, 970 Taiwan; 10 Department of Neurosurgery, Hualien Tzu Chi Hospital, Hualien, 970 Taiwan; 11 Department of Pathology, Hualien Tzu Chi Hospital and Tzu Chi University, Hualien, 970 Taiwan; 12 Department of General Dentistry, Linkou Chang Gung Memorial Hospital, Taoyuan City, 333 Taiwan; 13 School of Dentistry, College of Oral Medicine, Taipei Medical University, Taipei, 110 Taiwan; 14 Graduate Institute of Dental and Craniofacial Science, College of Medicine, Chang Gung University, Taoyuan City, 333 Taiwan; 15 Organ Transplantation Center, China Medical University Hospital, Taichung, 404 Taiwan

**Keywords:** Induced pluripotent stem cells, Neural stem cells

## Abstract

Stem cells have the potential to replace damaged or defective cells and assist in the development of treatments for neurodegenerative diseases, including Parkinson’s disease (PD) and Alzheimer’s disease. iPS cells derived from patient-specific somatic cells are not only ethically acceptable, but they also avoid complications relating to immune rejection. Currently, researchers are developing stem cell-based therapies for PD using induced pluripotent stem (iPS) cells. iPS cells can differentiate into cells from any of the three germ layers, including neural stem cells (NSCs). Transplantation of neural stem cells (NSCs) is an emerging therapy for treating neurological disorders by restoring neuronal function. Nevertheless, there are still challenges associated with the quality and source of neural stem cells. This issue can be addressed by genetically edited iPS cells. In this study, shRNA was used to knock down the expression of mutant α-synuclein (SNCA) in iPS cells that were generated from SNCA A53T transgenic mice, and these iPS cells were differentiated to NSCs. After injecting these NSCs into SNCA A53T mice, the therapeutic effects of these cells were evaluated. We found that the transplantation of neural stem cells produced from SNCA A53T iPS cells with knocking down SNCA not only improved SNCA A53T mice coordination abilities, balance abilities, and locomotor activities but also significantly prolonged their lifespans. The results of this study suggest an innovative therapeutic approach that combines stem cell therapy and gene therapy for the treatment of Parkinson’s disease.

## Introduction

Parkinson’s disease (PD) is the most common form of Parkinsonism and is characterized by reduced levels of dopamine (DA) and its metabolites (homovanillic acid [HVA]) in the striatum and pallidum [1]. Progressive loss of degenerating neuromelanin (NM)-containing dopaminergic neurons from the substantia nigra (SN) and Lewy bodies has also been observed in Parkinsonian midbrains [2, 3]. PD is a heterogeneous disease, and its treatments can be divided into two approaches: pharmacologic approaches (e.g., levodopa and DA agonists) and nonpharmacologic approaches (e.g., exercise and physical therapies) [4]. However, these therapies only alleviate the symptoms of PD and may entail debilitating side effects. Additionally, these therapies lose efficacy over time, as they do not modify the progression of PD [5]. Most cases of PD are sporadic, whereas 10–15% of PD patients account for familial (genetic) forms of PD [6]. Mutations in genes, including α-synuclein (SNCA) [7], leucine-rich repeat serine/threonine kinase 2 [8], Parkin [9], and DJ-1 [10] are reportedly associated with PD. Either missense mutation of SNCA or multiplication of the normal SNCA can result in parkinsonism and subsequent dementia [11, 12]. In addition, autosomal dominant Parkinson’s disease can be caused by duplications of the SNCA gene [13]. The importance of SNCA in the pathogenesis of familial PD has been reported by the presence of several missense mutations, e.g., A30P, E46K, H50Q, G51D, and A53T [14]. Additionally, triplication of the SNCA locus has been reported to cause Parkinson’s disease [12]. The B6.Cg-Tg(THY1-SNCA*A53T)F53Sud/J mice (SNCA A53T transgenic mice) have been established as a valuable model for the study of PD [15]. These transgenic mice harbor the mutated human SNCA gene driven by the human thymus cell antigen 1 theta (THY1) promoter, resulting in high levels of expression. The transgene expression is increased 10-fold in the brain and 20-fold in the spinal cord. SNCA A53T transgenic mice spontaneously developed Parkinson-like phenotypes, including progressive motor deficits, the presence of intraneuronal inclusion bodies, and neural cell loss upon aging. Moreover, the brain regions of SNCA A53T transgenic mice show SNCA-dependent neural degeneration associated with increased SNCA aggregation. Compared to the wild-type, SNCA A53T transgenic mice display significantly greater neurotoxicity. In brief, the human SNCA A53T transgenic mice provide a useful animal model for studying familial PD, which is caused by genomic multiplications of SNCA.

In recent years, stem cell therapy has provided new medical treatments for PD [16]. Embryonic stem (ES) cells and induced pluripotent stem cells can differentiate into cells of the three germ layers, indicating their ability to produce healthy cells for therapeutic purposes. Despite the application of ES cells as therapeutic approaches in animal models [17–19], there are still two important roadblocks that must be overcome: post-transplantation immune rejection and ethical issues before their application in humans. The induced pluripotent stem (iPS) cell is a novel research field for exploring the therapeutic potential of stem cells [20]. They resemble ES cells in morphology, gene expression, proliferation, surface antigens, epigenetic status of pluripotent cell-specific genes, and telomerase activity [21]; however, iPS cell technology has addressed both concerns: iPS cells can be generated from patient’s somatic cells (thus eliminating the potential for immune rejection and ethical considerations). Furthermore, patient-specific iPS cells can be used for drug screening and regenerative medicine research.

Human fetal neural stem cells have shown significant promise in treating Parkinson’s disease [22]. Despite this, the quality and source of neural stem cells remain challenging, whereas iPS cells are expected to serve as an alternative source of cells for PD. However, iPS cells reprogrammed from somatic cells carrying mutations in the SNCA gene inherit the same mutations. Consequently, NSCs derived from these iPS cells are also characterized by the same mutations that compromise their therapeutic potential.

Here, we describe a novel therapeutic approach for the treatment of familial PD. We collected the fibroblasts from SNCA A53T mice and reprogrammed them into iPS cells. We then generated iPS-shSNCA cells by knocking down the expression of mutated SNCA in these iPS cells. Finally, we differentiated iPS-shSNCA cells into neural stem cells (NSCs) and transplanted these NSCs into cortical areas of SNCA A53T transgenic mice to evaluate the therapeutic effects of NSCs.

## Results

### Generation and validation of the iPS cells

**Fig. 1:**
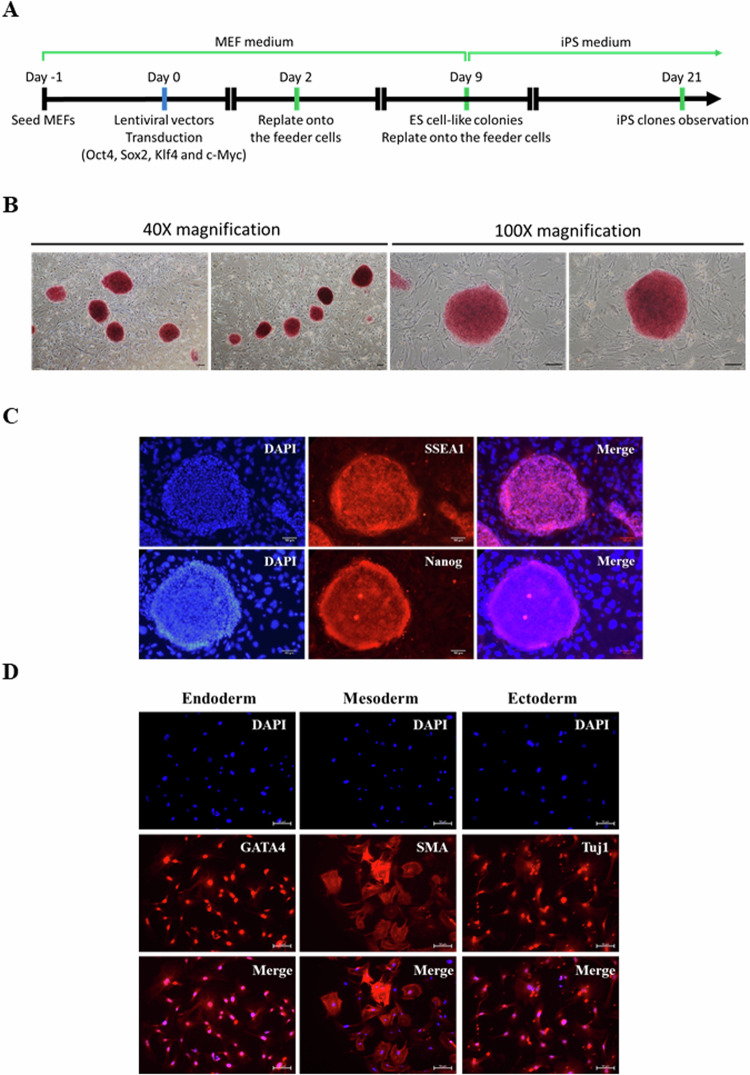
Generation of iPS cells from mouse embryonic fibroblasts. **A** Schematic illustration of the iPS cell reprogramming protocol. MEFs isolated for SNCA A53T mice were transduced with lentiviral vectors encoding Yamanaka factors. Afterward, the ES cell-like colonies were seeded onto mitomycin C-treated feeder cells and maintained in an iPS medium. **B** Representative images of AP-positive iPS colonies. The photos were documented by a phase-contrast microscope at 40× and 100× magnification, respectively. Scale bar: 200 μm. **C** Immunofluorescent staining of pluripotency markers SSEA1 and Nanog in iPS cells (red: SSEA1 and Nanog). Nuclei were stained with DPAI (blue). Digital images were taken with a fluorescence microscope (scale bar: 100 μm). **D** Analysis of the iPS cells for differentiation potential. The iPS cells were induced to spontaneously differentiate into three germ layers through embryoid bodies (EBs) formation. Differentiated cells expressed the markers for endoderm (GATA4), mesoderm (SMA), and ectoderm (Tuj1). Nuclei were stained with DPAI (blue). Digital images were taken with a fluorescence microscope (scale bar: 50 μm).

We collected the MEFs from SNCA A53T transgenic mice (B6.Cg-Tg(THY1-SNCA*A53T)F53Sud/J) according to the protocol described in the Materials and Methods section. These MEFs were transduced with lentiviral vectors encoding Oct4, Sox2, Klf-4, and c-Myc (Fig. 1A). Two days later, these cells were replated onto feeder cells, and the medium was refreshed every 2 days. We observed ES cell-like colonies and replated these cells onto feeder cells on day 9. After seeding, the medium was changed to iPSC culture medium, and these cells were subcultured every 7 days. We obtained several colonies with ES cell-like morphology on day 21 (12 days after replating on feeder cells). After passaging these clones and seeding them on new feeder cells (>10 passages), the iPS cells maintained ES cell-like morphologies. Further, these iPS cells stained strongly positive for alkaline phosphatase activity (Fig. 1B) and stem cell markers (SSEA1 and Nanog) as determined by AP kit and immunofluorescent staining, respectively (Fig. 1C). Next, we generated embryoid bodies (EBs) from these iPS cells and plated these EBs on gelatin-coated culture for spontaneous differentiation to examine their pluripotency. We observed heterogeneous cell populations growing out of the EBs after 6 days of culture. As shown in Fig. 1D, the differentiation potential of the generated iPS cells was confirmed through the detection of immunofluorescence positivity of GATA-binding protein 4 (GATA4) in the endodermal differentiation, smooth muscle actin (SMA) in the mesodermal differentiation, and beta-III tubulin (Tuj1) in the ectodermal differentiation. Together, these data revealed the functional pluripotency of the iPS cells and provided evidence of the successful generation of iPS cells.

### Differentiation of the iPS cells into NSCs

**Fig. 2:**
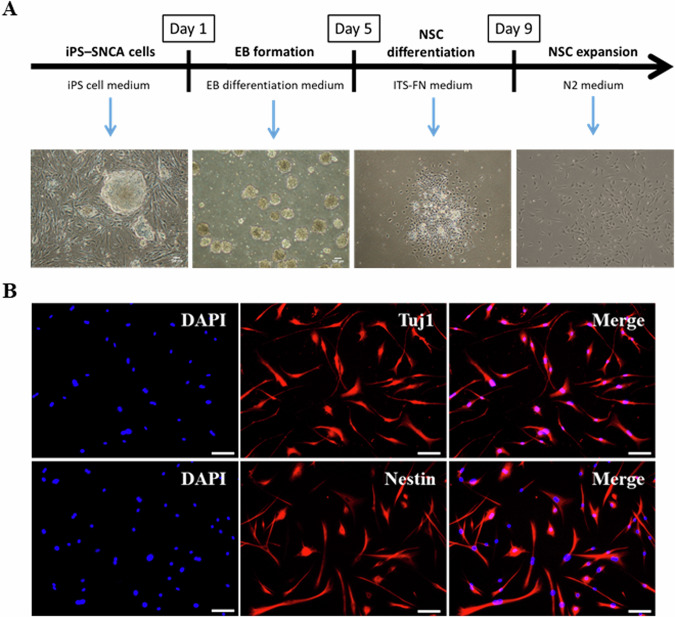
Differentiation of the iPS cells into neural stem cells. **A** Schematic illustration of the neural stem cell differentiation protocol. Digital images were taken with a phase-contrast microscope (scale bar: 100 μm). **B** Immunofluorescence staining for NSC markers Tuj1 (red, upper panel) and Nestin (red, lower panel) in NSCs. Nuclei were stained with DPAI (blue). Digital images were taken with a fluorescence microscope (scale bar: 20 μm).

After generating and characterizing the iPS cells, we explored their ability to differentiate into NSCs through embryoid bodies (Fig. 2A). The results showed that the iPS cells, morphology in the iPS cell medium were compact colonies that have distinct borders and well-defined edges (Fig. 2A, left panel). At the EB differentiation medium, the iPS cells formed floating spheroid structures (Fig. 2A, second image from the left). After plating the EBs onto poly-D-lysine coated plates, cells were cultured in ITS-FN medium for 4 days (Fig. 2A, second image from the right). Finally, After several passagings (2–3 passages), the differentiated cells showed distinct NSC morphology (Fig. 2A, right panel). We characterized the iPS cells-derived NSCs regarding the expression of typical markers. Most of the cells were positive for NSC markers Tuj1 (Fig. 2B, upper panel) and Nestin (Fig. 2B, lower panel). This indicated that the cell retained the phenotype of NSCs.

### Knockdown the expression of mutated SNCA by shRNAs

**Fig. 3:**
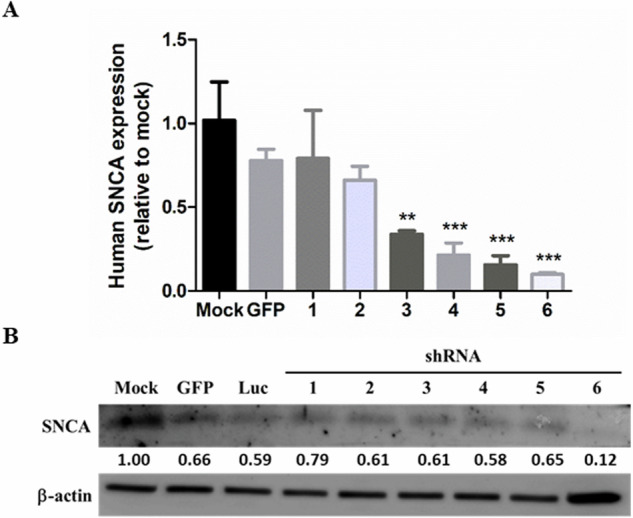
Evaluation of the knockdown efficiencies of SNCA shRNAs. **A** The expression levels of human SNCA mRNA in GBM8901 cells transfected with shRNAs against GFP and SNCA. The GBM8901 cells were transfected for 48 h and collected for real-time PCR analysis. The data were collected from at least three independent experiments. Bars represent mean and SD. Differences between the control group (mock) and experimental groups (GFP and shRNA-1 to shRNA-6) were evaluated by one-way analysis of variance and the Newman-Keuls Multiple Comparison Test. *P* < 0.05 indicates statistical significance (***P < 0.001). (shRNA-6 vs Mock, GFP, shRNA-1 and shRNA-2: *P* < 0.001; shRNA-5 vs Mock, shGFP, shRNA-1 and shRNA-2: *P* < 0.001; shRNA-4 vs Mock, GFP, shRNA-1 and shRNA-2: *P* < 0.001; shRNA-3 vs Mock GFP and shRNA-1: *P* < 0.001; shRNA-3 vs shRNA-2: *P* < 0.01; shRNA-2 vs Mock: *P* < 0.05). **B** Representative western blot images of SNCA in GBM8901 cells. Western blot analyses of human SNCA in GBM8901 cells following transfection with either shRNA against GFP, luciferase, or SNCA. The shRNAs against GFP and luciferase were used as negative controls. The signal was quantified by ImageJ software.

We investigated the knockdown efficiency of six shRNAs against human SNCA in the GBM8901 glioma cell line. The shRNA sequences are listed in Table 1. There were six shRNAs against SNCA, but only shRNA-3 to shRNA-6 efficiently knocked down SNCA mRNA expression (Fig. 3A). The expression of the SNCA protein was also investigated in GBM8901 cells transfected with shRNAs against GFP, Luc, and SNCA. shRNA-6 exhibited the strongest inhibitory effect on SNCA protein expression, as shown in Fig. 3B. Overall, shRNA-6 exhibited potent inhibition of SNCA expression (∼90%). Further experiments were performed using shRNA-6. Next, we co-transfected 293T cells with the shRNA-6 plasmid and lentiviral package vectors to generate lentiviral particles. SNCA knockdown iPS cells (iPS-shSNCA cells) were established by incubating the iPS cells with shRNA-6 lentivirus. Subsequently, iPS and iPS-shSNCA cells were differentiated into NSCs and NSC-shSNCA cells according to the aforementioned protocol for further studies.

**Table 1:** The sequence information of shRNAs.

	Gene symbol	Target sequence	Oligo sequence
1	SNCA	ACCAAAGAGCAAGTGACAAAT	CCGGACCAAAGAGCAAGTGACAAATCTCGAGATTTGTCACTTGCTCTTTGGTTTTTT
2	SNCA	TGACAATGAGGCTTATGAAAT	CCGGTGACAATGAGGCTTATGAAATCTCGAGATTTCATAAGCCTCATTGTCATTTTTG
3	SNCA	ACCAAAGAGCAAGTGACAAAT	CCGGACCAAAGAGCAAGTGACAAATCTCGAGATTTGTCACTTGCTCTTTGGTTTTTTG
4	SNCA	AGGACCAGTTGGGCAAGAATG	CCGGAGGACCAGTTGGGCAAGAATGCTCGAGCATTCTTGCCCAACTGGTCCTTTTTTG
5	SNCA	CTGACAATGAGGCTTATGAAA	CCGGCTGACAATGAGGCTTATGAAACTCGAGTTTCATAAGCCTCATTGTCAGTTTTT
6	SNCA	GAAGCCTAAGAAATATCTTTG	CCGGGAAGCCTAAGAAATATCTTTGCTCGAGCAAAGATATTTCTTAGGCTTCTTTTTG
	GFP	AAGCTGACCCTGAAGTTCAT	CCGGCAAGCTGACCCTGAAGTTCATCTCGAGATGAACTTCAGGGTCAGCTTGTTTTTG
	Luciferase	CTTCGAAATGTCCGTTCGGTT	CCGGCTTCGAAATGTCCGTTCGGTTCTCGAGAACCGAACGGACATTTCGAAGTTTTTG

### Evaluation of the effects of stem cell therapy in PD mouse model

**Fig. 4:**
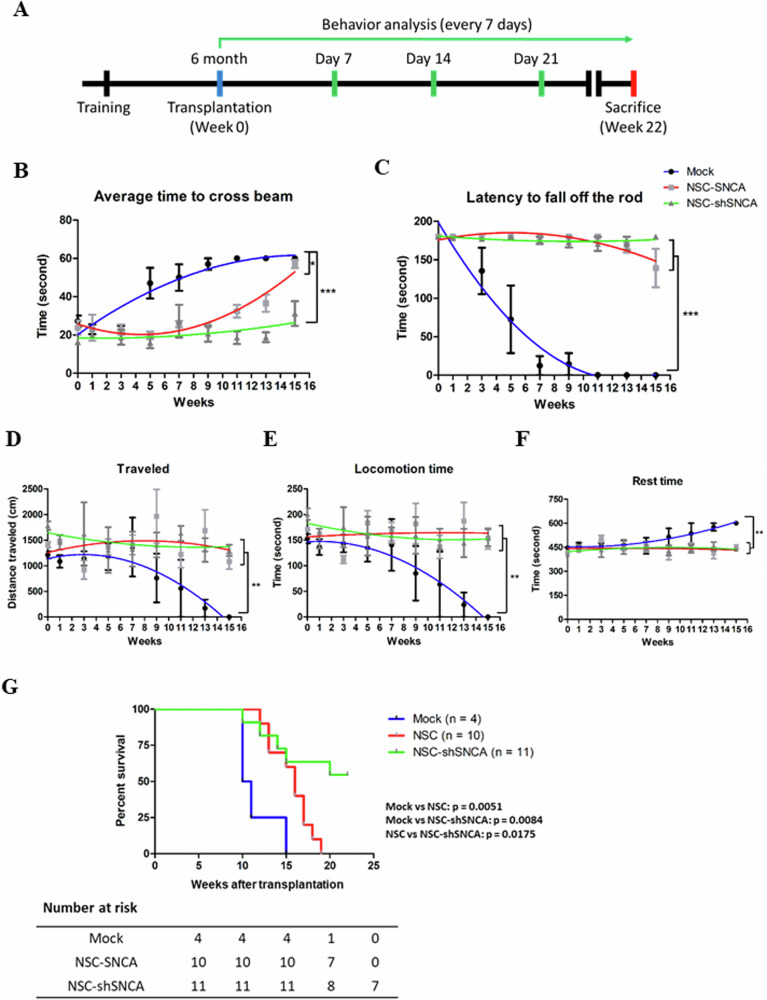
Exploration of the therapeutic effects of NSCs and NSC-shSNCA cells on behavioral tests. **A** Schematic illustration of the behavior analysis protocol. At the age of 5 months, six training sessions were conducted for SNCA A53T transgenic mice over 2 weeks. Following the last training, mice were transplanted with NSCs or NSC-shSNCA cells. In a control group (mock), mice were injected with normal saline. NSCs and NSC-shSNCA cells were assessed for their therapeutic effects by **B** beam walking (mock vs NSC: week 15 to week 13, *P* < 0.001; mock vs NSC-shSNCA: week 7, *P* < 0.01, week 5 and 9 to 15, *P* < 0.001; NSC vs NSC-shSNCA: week 13, *P* < 0.05, week 15, *P* < 0.001), **C** rotarod (mock vs NSC: week 5 to week 15, *P* < 0.001; mock vs NSC-shSNCA: week 5 to week 15, *P* < 0.001; NSC vs NSC-shSNCA: NS), **D** total traveled distance (mock vs NSC: week 13, *P* < 0.05; mock vs NSC-shSNCA: NS; NSC vs NSC-shSNCA: NS), **E** locomotion time (mock vs NSC: week 13, *P* < 0.01, week 15, *P* < 0.05; mock vs NSC-shSNCA: week 15, *P* < 0.01; NSC vs NSC-shSNCA: NS), and **F** rest time (mock vs NSC: week 13 and 15, *P* < 0.01; mock vs NSC-shSNCA: week 15, *P* < 0.01; NSC vs NSC-shSNCA: NS). The behavioral assessment was conducted 1 day (week 0) before transplantation and every 7 days thereafter for 22 weeks. Data are presented as mean ± SEM values (**B**–**F**). Behavioral data were compared by two-way analysis of variance (ANOVA) for time and treatment effects followed by a post hoc Bonferroni test (corrected for multiple comparisons). **G** Overall survival curves for transplanted mice in the behavior tests. Survival analysis was done using the Kaplan-Meier estimator and the log-rank test for group comparison. Variables with a significant *P*-value in the univariate analysis were exposed to a multivariate analysis using Cox regression proportional hazard model. GraphPad Prism 5.01 (San Diego, CA, USA) was used for analysis with a significance level of *P* < 0.05. NS stands for not statistically significant.

To assess the functional recovery of PD model mice with transplanted NSCs and SNCA-shSNCA cells, the mice were trained according to the protocol described in Fig. 4A. We transplanted 1 × 10^6^ of NSCs or NSC-shSNCA cells into the brain of SNCA A53T mice and performed the behavior analysis every 7 days. Since death typically occurs within 1–2 weeks after disease onset (around 8 months after birth) and treatment is not expected to be effective, administration is initiated before the onset (6 months after birth). In beam walking (Fig. 4B), rotarod (Fig. 4C), and locomotor activity experiments (Fig. 4D–F), mice treated with normal saline (mock) demonstrated significant impairments in balance, coordination, and motor skills upon aging. As a comparison, mice transplanted with NSCs or NSC-shSNCA cells exhibited significant improvement in their balancing, coordination, and motor skills. We observed no significant differences between the NSC and NSC-shSNCA groups in the beam-walking, rotarod, and locomotor activity experiments. However, the mice treated with NSC-shSNCA cells showed prolonged lifespans compared to those of the NSC and mock groups (Fig. 4G). To our surprise, more than 60% of mice survived at the end of the experiment (week 22 after the operation), whereas the last mice in the mock and SNCA groups died at 16 and 20 weeks after the operation, respectively.

### DA neuron and apoptotic cell populations in PD mouse brains

**Fig. 5:**
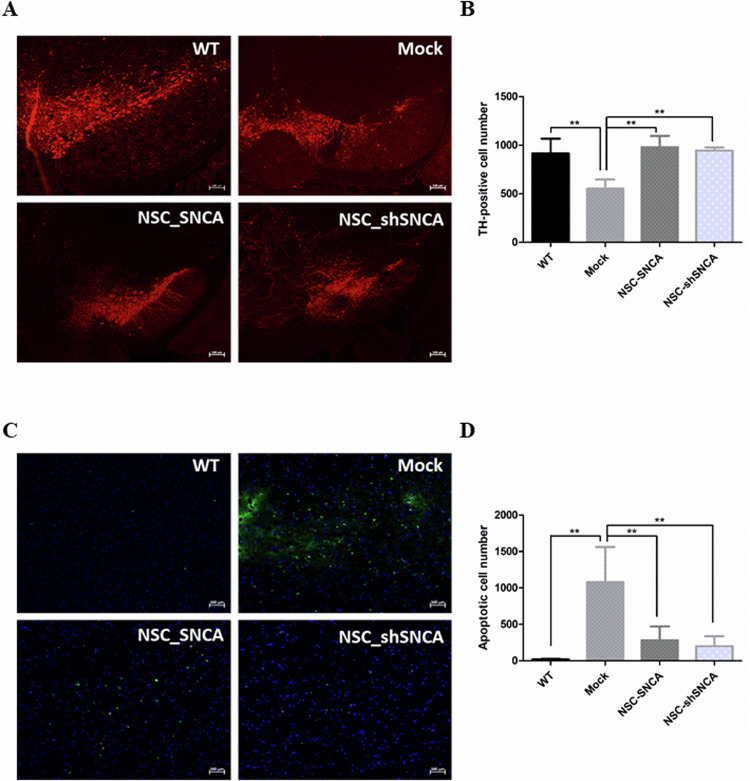
Exploration of the TH-positive and apoptotic cells in PD mice brains. **A** Representative images of TH-positive cells in the SN region. Brain samples were collected from C57BL/6J mice (WT) and mice in the behavior tests (mock, NSC, and NSC-shSNCA). Frozen sections of the SN region were prepared and stained with the dopaminergic neuron marker, TH (red). **B** The TH-positive cells were quantified by ImageJ software. At least three sections in each group were evaluated. One-way ANOVA followed by the Newman-Keuls test for equal variances was used to evaluate the differences. **C** Representative images of apoptotic cells in the SN region. Frozen sections of the SN region from the aforementioned samples were stained with a TUNEL assay kit. The apoptotic cells were visualized by green fluorescence. **D** The apoptotic cells were quantified by ImageJ software. At least three sections in each group were evaluated. One-way ANOVA followed by the Newman-Keuls test for equal variances was used to evaluate the differences. Bars represent mean and SD. GraphPad Prism 5.01 (San Diego, CA, USA) was used for analysis with a significance level of *P* < 0.05 (**P < 0.01).

To verify the successful transplantation, the brains of each group of mice in the behavior experiment were collected and preserved in paraformaldehyde. The samples were stained for the dopaminergic neuron marker tyrosine hydroxylase (TH) (Fig. 5A). Our results indicated that TH-positive neurons in the substantia nigra of SNCA A53T transgenic mice (mock) were significantly fewer than those in wild-type mice and NSC-transplanted mice (NSC and NSC-shSNCA). It is important to note, however, that the NSC and NSC-shSNCA groups were not significantly different (Fig. 5B). Simultaneously, we explored apoptotic cells in the same area in each group with TUNEL staining (Fig. 5C). Although the number of apoptotic cells in the substantia nigra was dramatically increased in SNCA A53T transgenic mice, NSC transplantation exhibited obvious countereffects in both the NSC and NSC-shSNCA groups. Nonetheless, the NSC-SNCA and NSC-shSNCA groups exhibited no significant differences (Fig. 5D).

## Discussion

In addition to their self-renewal abilities, NSCs are considered potential grafts for cellular transplantation because of their ability to differentiate into all neural-lineage cells, including neurons, astrocytes, and oligodendrocytes. For tissue restoration in PD, NSC-derived neurons, astrocytes, and oligodendrocytes can form functional neurovascular units in conjunction with endothelial cells and pericytes [23]. Human fetal neural stem cells have shown significant promise in treating Parkinson’s disease [22]. Despite this, the availability of high-quality neural stem cells and the source of such cells remain challenging. iPS cells, on the other hand, are expected to provide PD patients with an alternative source of cells. Patients suffering from Parkinson’s disease have shown therapeutic benefits from personalized iPSC-derived dopaminergic progenitor cells [24]. In addition, the patient treated with personalized iPSC-derived dopamine progenitor cells showed stabilized or improved symptoms of PD [25]. There is a risk that using autologous cells from a PD patient who suffers from genetic defects may prevent therapeutic efficacy due to mutant SNCA, leucine-rich repeat serine/threonine kinase 2, Parkin, or DJ-1 genes. In this study, we investigated the therapeutic effect of NSCs derived from SNCA knockdown-iPS cells and found that the transplantation of NSCs not only improved mice’s coordination abilities, balance abilities, and locomotor activities but also significantly prolonged their lifespans.

We observed that the SNCA A53T mice exhibited improved balance, coordination, and locomotion abilities following the NSC transplantation (Fig. 4B–F). However, the therapeutic effects did not significantly differ between the NSC and NSC-shSNCA groups. One possible explanation is that the pathophysiology of Parkinson’s disease is highly complex, where SNCA represents only one aspect. Even if SNCA expression is successfully inhibited, behavioral outcomes can still be significantly influenced by other factors, such as neurodegenerative changes, inflammatory responses, and neuronal dysfunction. Moreover, the shRNA-mediated suppression of SNCA expression may have exceeded its optimal time window by the time of transplantation, or the suppression effect may have been insufficient to demonstrate significant differences in behavioral tests. Following neural stem cell transplantation, pathological processes may have already exerted substantial effects on behavioral outcomes, surpassing the isolated impact of SNCA expression. We found that NSCs and NSC-shSNCA cells transplantation improved and ameliorated Parkinson’s symptoms. Additionally, NSCs or NSC-shSNCA cells transplantation exhibited similar effects on the elevation of TH-positive neurons and the reduction of apoptosis in mice (Fig. 5). These findings confirm previous reports that NSC transplantation can ameliorate SNCA-induced Parkinson’s disease [23].

The therapeutic effects of NSC transplantation in PD can be both directly and indirectly. NSCs can either directly enhance dopaminergic neuron differentiation, the release of dopamine, reinnervation of the striatum, or integration of neural circuits after transplantation or indirectly facilitate dopaminergic differentiation by secreting neurotrophic factors, including brain-derived neurotrophic factor (BDNF), nerve growth factor (NGF), cerebral dopamine neurotrophic factor (CDNF) and glial-derived neurotrophic factor (GDNF) [26]. The NSC transplantation in our animal model not only alleviated PD symptoms but also extended the survival of the animals. The mice transplanted with NSC-shSNCA cells had the longest life expectancy compared with those of the mice in the mock and NSC groups (Fig. 4G). As these NSCs still express mutant SNCA, supplementation with NSCs may improve PD symptoms, but cannot affect the progression of the disease. These NSC-shSNCA cells, on the other hand, might be a more suitable source of cells for treating Parkinson’s disease. According to the current state of research, a number of clinical trials have examined how stem cell-derived therapy can treat PD [27]. Various types of stem cells have been applied for cell-based therapy in PD, including fetal ventral mesencephalic Cells, ES cells, and iPS cells [27, 28]. The development of strategies for using well-educated and qualified stem cells as stem cell-derived therapeutics is urgently needed to improve the quality of life of patients.

Gene therapy and cellular engineering techniques have enhanced the therapeutic effects of NSC transplantations. For example, one study found that genetically edited human NSC overexpressing choline acetyltransferase (ChAT) could significantly better enhance cognitive function and physical activity in elderly animals [29]. Moreover, a recent study indicated that the transplantation with neural precursor cells derived from SUPT4H1 gene-edited iPS cells exhibited promising therapeutic effects in a mouse model for Huntington’s disease [30]. In PD, SNCA may serve as a valid target, and shRNA against SNCA may offer potential neuroprotective benefits [31]. In addition to SNCA, several genes were either upregulated (e.g., RAP1GA1, RIMS1, TP53, and NR2F2) or downregulated (e.g., AGTR1, PARK1, 5, 7, 9, and 10) in PD [32, 33]. Several strategies have been developed to silence SNCA, including RNA interference, which targets SNCA directly, or microRNAs, which target SNCA’s 3’-untranslated region of SNCA [34, 35]. In recent years, multiplexed CRISPR technology, as well as zinc-finger nucleases and transcription activator-like effector nucleases, have significantly expanded the possibilities of stem cell-derived therapies [36]. In this study, we demonstrated the potent effects of NSCs derived from mutant SNCA knockdown-iPS cells on treating PD and provided experimental evidence for further clinical application of iPS cells in cell-based therapeutics for PD. As multiple genes were altered in PD, NSCs derived from multiple gene-edited iPS cells may be more effective in treating PD.

**Fig. 6:**
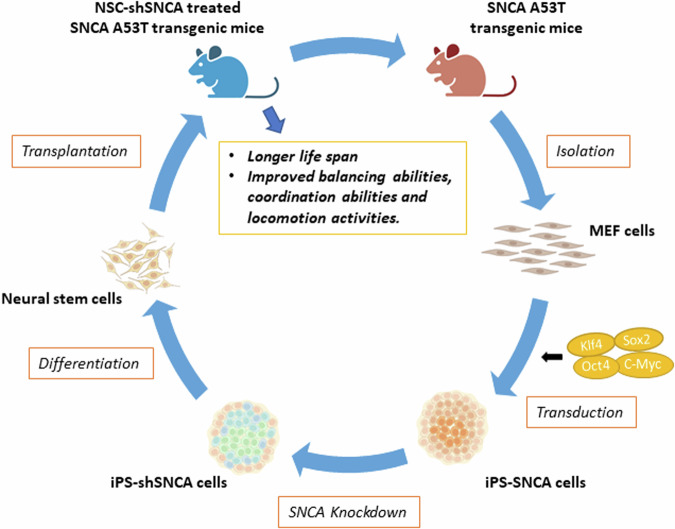
Schematic illustration of genetically edited iPS cell-derived NSCs rescue Parkinson’s disease. In this study, we isolated MEFs from SNCA A53T mice and reprogrammed these cells into iPS cells. After knocking down the expression of SNCA, these iPS cells were differentiated into NSCs and transplanted into SNCA A53T mice. Our animal study demonstrated that mice treated with NSCs exhibited improved balancing, coordination, and locomotion activities. Furthermore, the mice treated with NSC-shSNCA cells showed prolonged lifespans.

We demonstrated the therapeutic effects of NSCs derived from SNCA knockdown-iPS cells in a PD mouse model (Fig. 6). The transplantation of either NSCs or NSC-shSNCA cells into SNCA A53T transgenic mice dramatically improved their PD-like symptoms. Mice transplanted with NSC-shSNCA cells exhibited extended lifespans. Our data indicated that the transplantation of NSC-shSNCA cells could compensate for the loss of dopaminergic cells in these mice during aging without expressing SNCA, resulting in a prolonged lifespan. Collectively, our study has shown that the knockdown of mutant SNCA in iPS cells can provide a suitable source of NSCs for the treatment of Parkinson’s disease caused by mutations in SNCA.

## Materials and methods

### Mouse embryonic fibroblast cell isolation and animal model

We obtained C57BL/6J and SNCA A53T transgenic mice from the Jackson Lab (Bar Harbor, ME, USA), whose detailed information can be found at the following links (http://www.jax.org/strain/000664 and http://www.jax.org/strain/008859, respectively). These transgenic mice express human A53T variant SNCA under the promoter of human thymus cell antigen 1. Hind limb paralysis and a resting tremor typically begin to appear around 8 months of age. We isolated primary mouse embryonic fibroblast cells (MEFs) from SNCA A53T transgenic mice at 13.5 days old. During the procedure, the embryos were retrieved by cesarean section, freed from the placenta, and their internal organs, legs, and brains were removed. Trypsin-EDTA (GIBCO BRL, Grand Island, NY, USA) was used to digest the remaining embryo parts. Immediately following digestion, MEFs were cultured in MEF medium (DMEM/high glucose (GIBCO BRL) with 10% heat-inactivated FBS (HyClone, Logan, Utah, USA), penicillin (100 U/ml; GIBCO BRL), streptomycin (100 µg/ml; GIBCO BRL), nonessential amino acids (0.1 mM, GIBCO BRL) and L-glutamine (2 mM, GIBCO BRL)) in a humidified incubator with 5% CO_2_. The Institutional Animal Care and Use Committee at China Medical University approved all experimental protocols (CMUIACUC-2017-313).

### The generation of iPS cells

On day 0, SNCA A53T MEFs were transduced with lentiviral vectors expressing mouse Oct4, Sox2, Klf4, and c-Myc (Cellexium Biomedica Inc. Taipei, Taiwan). ES-like colonies were found on day 9, and these cells were transferred to feeder cells in an iPS cell culture medium consisting of DMEM/high glucose with 15% heat-inactivated defined FBS (HyClone, Logan, Utah, USA), penicillin (100 U/ml), streptomycin (100 µg/ml), nonessential amino acids (0.1 mM), L-glutamine (2 mM), 2-Mercaptoethanol (0.1 mM) (Merck KGaA, Darmstadt, Germany), and mouse Leukemia Inhibitory factor (LIF, 10^3^ units/ml; EMD Millipore Corporation, Temecula, California, USA). On day 21, iPS cells (ES cell-like clones) were obtained.

### Alkaline phosphatase staining and indirect immunofluorescent staining

The alkaline phosphatase (AP) staining procedure (Vector Laboratories, Cat. No. SK-5100, Newark, California, USA) was followed according to the manufacturer’s instructions. We treated the cells at room temperature with 0.5% Triton X-100 for 15 min after fixing them with 4% paraformaldehyde in PBS for 30 min. After washing with PBS, cells were treated with blocking buffer (0.5% BSA in PBS) for 1 h at room temperature and washed 3 times with PBS. We used the following primary antibodies at 1:1000 dilutions overnight at 4 °C: anti-Nanog (Genetex, Cat. No. GTX100863, Irvine, CA, USA), anti-SSEA1 (Millipore, Cat. No. MAB4301, Burlington, MA, USA), anti-GATA4 (Genetex, Cat. No. GTX113194), anti-SMA (Millipore, Cat. No. CBL171), anti-Tuj-1 (Millipore, Cat. No. MAB1637) and anti-Nestin (Abcam, Cat. No. ab6142, Cambridge, UK). Cells were washed with three cold PBS washes and then incubated with FITC-conjugated anti-mouse IgG or TRITC-conjugated anti-rabbit IgG (Sigma-Aldrich, Cat. No. AP160F and T6778). A confocal fluorescence microscope (TCS-NT, Hilden, Germany) was used to visualize the signals. Cell nuclei were stained with DAPI (Sigma-Aldrich).

### Histological analysis of spontaneous differentiation in vitro

The iPS cells were seeded into bacterial culture dishes at a density of 5 × 10^6^ cells/ml in iPS cell culture media without LIF for 72 h, and the aggregated cells (embryoid bodies, EBs) were then plated onto gelatin-coated culture plates for another 3 days. A confocal fluorescence microscope was used to observe the signals of spontaneously differentiated cells stained with anti-GATA4, anti-SMA, and anti-Tuj1.

### NSC differentiation from iPS cells

On day 1, we dissociated iPS cells into single cells using accutase (Thermo Fisher Scientific, Waltham, MA, USA), then transferred them to bacterial culture dishes containing EB differentiation media (DMEM supplemented with 20% FBS (Gibco), glutamine (2 mM), nonessential amino acids (0.1 mM), and 2-mercaptoethanol). Upon renewing the culture medium every 2 days, iPS cells differentiated spontaneously into EBs. EBs were replated on day 5 with poly-D-lysine coated plates (Gibco). By switching the medium to ITS-FN medium (DMEM/F12, glutamine (2 mg), 1X ITS-G supplement (Gibco), Fibronectin (5 µg/ml, Gibco), 2-mercaptoethanol (0.1 mM)), neural stem cells were produced. After 4 days, cells were washed with PBS and dissociated with trypsin-EDTA for 5 min. The cell pellets were resuspended in N2 medium (DMEM/F12, glutamine (2 mM), 1X N2 supplement (Gibco), and bFGF (20 ng/ml, Gibco)) and seeded onto Poly-L-ornithine/Fibronectin-coated cultureware following brief centrifugation. NSCs could be maintained for five passages, and the culture medium was replaced every 2 days.

### Efficacy of shRNAs in knocking down SNCA genes

We examined the knockdown efficiency of different shRNAs in GBM8901 glioma cells in this study. The cells were cultured in RPMI-1640 medium containing FBS, nonessential amino acids, glutamine, penicillin, and streptomycin. According to the manufacturer’s instructions, shRNA encoded in plasmids was transfected into GBM8901 cells using FuGENE (Promega, Madison, WI, USA). SNCA gene expression levels were determined using real-time PCR using the delta-delta Ct method.

### Real-time PCR and Western Blot

RNA was collected from GBM8901 cells transfected with shRNA using Trizol reagent (Thermo Fisher Scientific). We used Fast SYBR TM Green Master Mix (Applied Biosystems, Waltham, MA, USA) and StepOnePlus TM Real-Time PCR Environment (Applied Biosystems) to measure gene expression (i.e., Actin and SNCA). According to the manufacturer’s instructions, 1 mg of total RNA was reverse transcribed-PCR using Maxima H Minus First Strand cDNA Synthesis Kit (Thermo Fisher Scientific) and oligo(dT) primers in a final volume of 20 ml. Real-time PCR reactions were conducted following the manufacturer’s standard PCR protocol. Gapdh was used as a reference gene for normalizing mRNA expression levels. Negative controls were siRNAs against GFP. GBM8901 cells transfected with shRNA were lysed, and proteins were collected for Western blot analysis. Antibodies against SNCA (Novus Biologicals, Cat. No. NBP2-15365) and β-actin (Genetex, Cat. No. GTX629630) were used in this study.

### The production and transduction of shRNA-6 lentiviruses

293T cells were co-transfected with shRNA-6 plasmids, pCMV-DR8.91, and pMD.G using FuGENE. After transfection, 293T cells were cultured for 24 h with FBS, NEAA, L-glutamine, and PS in DMEM/high glucose medium, followed by 24 h in ITS-FN medium. The virus supernatant was collected, filtered through a 0.45 mm filter, and stored at −80 °C until the next step. For transduction, the iPS cells were incubated with virus solution for 24 h (MOI:5), followed by selection with 0.5 mg/ml of puromycin for another 24 h.

### Neurological behavioral measurements after transplantation

For the SNCA A53T transgenic mice, six training sessions were conducted over 2 weeks at the age of 5 months. After the last training session, mice were transplanted with 1 × 10^6^ NSC-SNCA or NSC-shSNCA cells, which were counted using the Countess 2 (Thermo Fisher Scientific), into three cortical areas adjacent to the right middle cerebral artery (MCA), positioned 3 to 3.5 mm below the dura after ligation [37]. Normal saline injections were administered as a control group for mice. According to “Methods of behavior analysis in neuroscience” [38], locomotor activity, beam walking, and rotarod were used to assess the therapeutic effects of NSCs and NSC-shSNCA cells. Briefly, the neuronal behaviors of target mice were examined using 3 separate apparatuses: a beam walking was used to monitor balancing ability (width: 80 cm), a rotarod was used to monitor coordination, and a locomotor device was used to monitor overall activity. During this study, mice’s balance ability was measured by recording their time crossing an 80-cm beam and measuring the frequency with which their rear feet slipped. During 3 min, animals were observed to remain stable on a rota. An 8-channel locomotor activity box was used to monitor overall activity for 1 h. The movement of the animals in the chamber, their resting time, and the distance they traveled during the last 30 min were also examined. The behavioral assessments were conducted 1 day before transplantation (week 0) and every 7 days thereafter for 22 weeks. Afterward, the mice were sacrificed, and their brain tissue was collected.

### Assessment of the cell numbers of dopaminergic neurons

We prepared frozen sections of brain tissue after the behavioral study. A dopaminergic cell marker, tyrosine hydroxylase (TH; Millipore), was stained in sections of the substantia nigra (SN). The number of TH-positive cells was determined using ImageJ software (National Institutes of Health, USA).

### TUNEL assay

An apoptotic cell count was determined using ImageJ software by staining the frozen sections with the TUNEL assay kit (Abcam, Cat. No. ab66108).

### Statistical analysis

Data were obtained from at least three independent experiments and compared for statistical significance using the Prism software (version 5.01, San Diego, CA, USA) at a significance level of *P* < 0.05 (*P < 0.05, **P < 0.01, and ***P < 0.001). One-way analysis of variance, followed by the appropriate multiple comparisons test, was used to investigate statistical significance. Data were expressed as the mean ± SD. Behavioral data were compared by two-way analysis of variance (ANOVA) followed by a post hoc Bonferroni test. Data were expressed as the mean ± SEM. Survival analysis was done using the Kaplan-Meier estimator and the log-rank test for group comparison. Variables with a significant *P*-value in the univariate analysis were exposed to a multivariate analysis using Cox regression proportional hazard model.

## Supplementary information

Original Data File

## Data Availability

All of the data used to support the findings of this study are included in the article.
