# Supplementary figures and images for: Neural stem cells derived from α-synuclein-knockdown iPS cells alleviate Parkinson’s disease
Source: Cell Death Discov. 2024 Sep 17;10:407. doi: 10.1038/s41420-024-02176-z (PMC11405526; doi:10.1038/s41420-024-02176-z)

SNCA

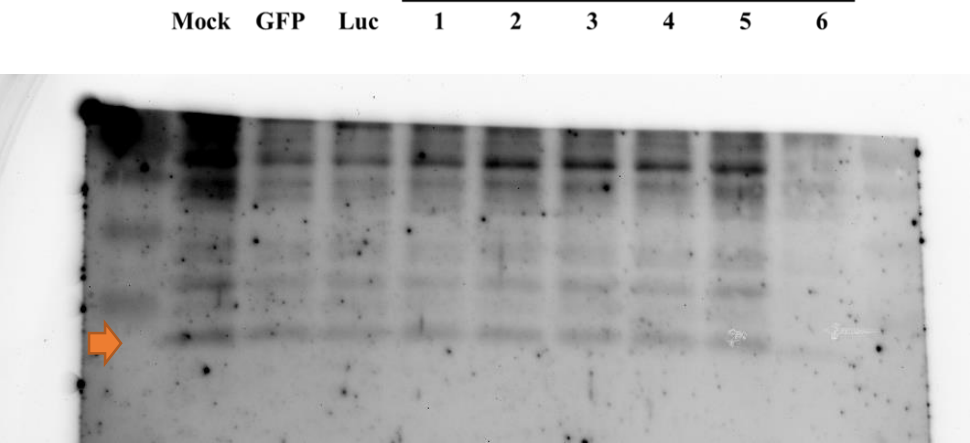

Mock shGFP shLUC

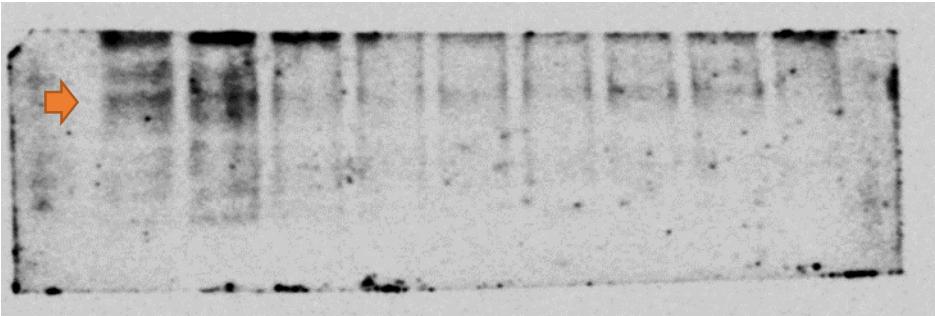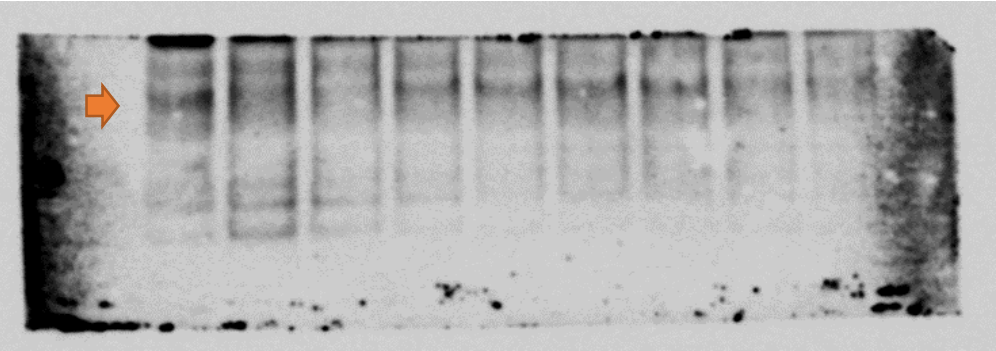

alpha-Synuclein Antibody (NBP2-15365), 14kDa

actin

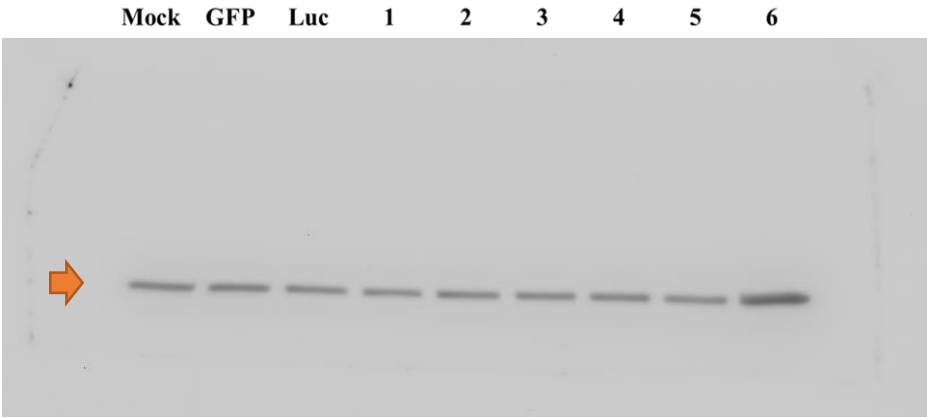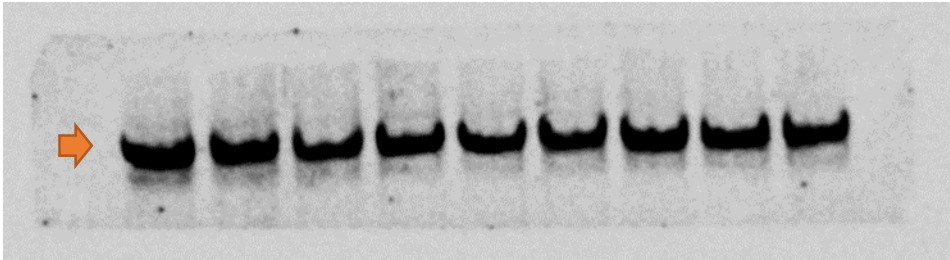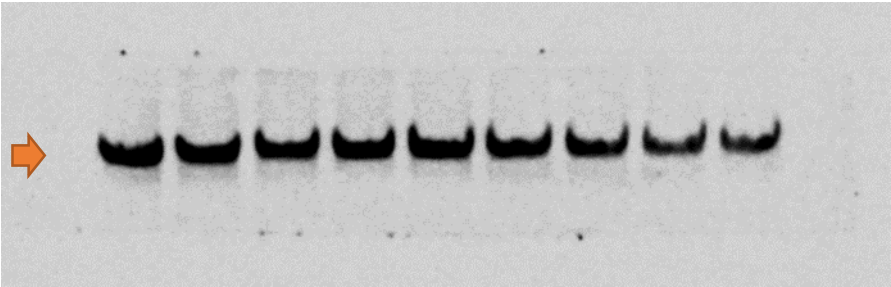

Beta actin (GTX 629630), 42kDa

Supplement: Supplementary file 1 — Original Data File [file 41420_2024_2176_MOESM1_ESM.pdf]
